# Supplementary material for: Activation of Soluble Polysaccharides with 1-Cyano-4-Dimethylaminopyridine Tetrafluoroborate (CDAP) for Use in Protein–Polysaccharide Conjugate Vaccines and Immunological Reagents. III Optimization of CDAP Activation
Source: Vaccines (Basel). 2020 Dec 18;8(4):777. doi: 10.3390/vaccines8040777 (PMC7766920; doi:10.3390/vaccines8040777)
Supplement: Supplementary file 1 [file vaccines-08-00777-s001.pdf]

Activation of soluble polysaccharides with 1-cyano-4-dimethylaminopyridine tetrafluoroborate (CDAP) for use in protein-polysaccharide conjugate vaccines and immunological reagents. III. Optimization of CDAP activation

Andrew Lees [1], Jackson F. Barr [1,2] and Samson Gebretnsae [1]

[1]Fina Biosolutions LLC, 9430 Key West Ave, Suite 200, Rockville, MD 20850, USA;
ALees@Finabio.com, JBarr@som.UMaryland.edu, Samson@Finabio.com
[2]Current address: University of Maryland School of Medicine, Baltimore, MD 21201, USA.
*Correspondence: ALees@Finabio.com

Supplementary Materials

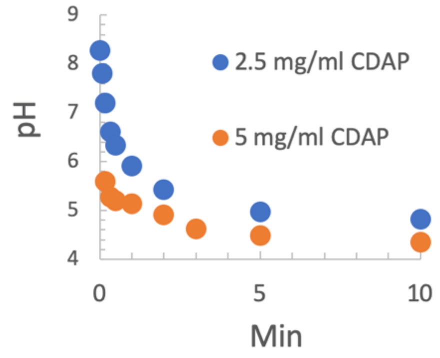

Figure S1. pH change of aqueous solution of CDAP. CDAP, from a 100 mg/ml solution in acetonitrile, was added to water to a final concentration of 2.5 or 5 mg/ml and the pH monitored.

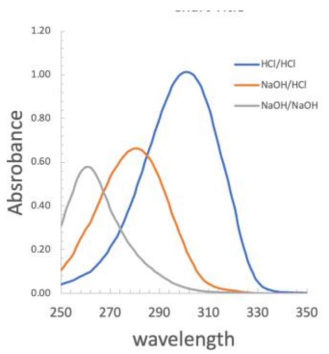

Figure S2. UV spectra of CDAP diluted into combinations of HCl and NaOH. 0.1M HCl/0.1 M HCl (blue), 0.1M NaOH/0.1 M HCl (orange), 0.1M NaOH/0.1 M NaOH (grey). The initial diluted concentration of CDAP was 35 μM.

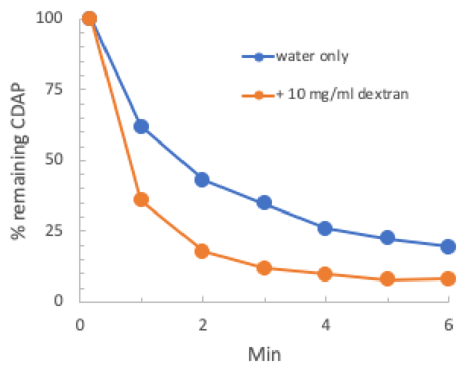

Figure S3. CDAP consumption in the presence and absence of 10 mg/ml dextran, pH 9 at 0° C. CDAP (10 mg/ml final) was added to 0.1 M sodium borate, pH 9 solution and aliquots periodically transferred to 0.1 M HCl CDAP consumption was monitored at 312 nm.

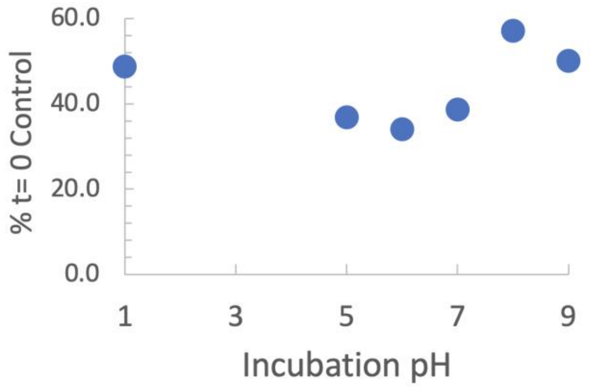

Figure S4. Stability of CDAP-activated dextran as a function of pH. Dextran was activated with CDAP at pH 9 and incubated at the indicated pH for two hours, on ice. 0.5 M ADH was then added. As the reaction of hydrazides with CDAP-activated dextran is essentially independent of pH[1], the pH was not further adjusted. Each sample was desalted after an overnight reaction and the ratio of hydrazide to dextran was determined. Results are expressed as percentage of a control in which the activated dextran was immediately added to the ADH solution. The following 0.1 M buffers were used: HCl (pH 1), sodium acetate (pH 5), MES (pH 6), HEPES (pH 7 and 8) and sodium borate (pH 9).

Diamine	Structure	NH_2 or Hz/dex
Ethylenediamine	$NH_2-(CH_2)_2-NH_2$	20
Propanediamine	$NH_2-(CH_2)_3-NH_2$	17
Butanediamine	$NH_2-(CH_2)_4-NH_2$	714
Hexanediamine	$NH_2-(CH_2)_6-NH_2$	854
Adipic dihydrazide	$NH_2NHC(O)-(CH_2)_4-C(O)NHNH_2$	808

Table S1. Functionalization of CDAP-activated dextran with diamines. 10 mg/ml T2000 Dextran was activated with 0.5 mg/mg CDAP using 0.1 M NaOH to maintain pH 9 for 15 min and aliquots combined with an equal volume of 0.5 M of each of the indicated diamines. Following an overnight reaction, the products were desalted and the amine or hydrazide to dextran ratio determined. The number of carbon and nitrogen atoms in the terminal diamine or dihydrazide is indicated.

1. Shafer, D. E.; Toll, B.; Schuman, R. F.; Nelson, B. L.; Mond, J. J.; Lees, A., Activation of soluble polysaccharides with 1-cyano-4-dimethylaminopyridinium tetrafluoroborate (CDAP) for use in protein-polysaccharide conjugate vaccines and immunological reagents. II. Selective crosslinking of proteins to CDAP-activated polysaccharides. *Vaccine* **2000,** *18* (13), 1273-81.
